# Construction of a high-density genetic map and the *X/Y* sex-determining gene mapping in spinach based on large-scale markers developed by specific-locus amplified fragment sequencing (SLAF-seq)

**DOI:** 10.1186/s12864-017-3659-9

**Published:** 2017-04-04

**Authors:** Wei Qian, Guiyan Fan, Dandan Liu, Helong Zhang, Xiaowu Wang, Jian Wu, Zhaosheng Xu

**Affiliations:** Institute of Vegetables and Flowers, Chinese Academy of Agricultural Sciences, Beijing, 100081 China

**Keywords:** *Spinacia oleracea* L, Genetic map, SLAF-seq, Sex-determining gene

## Abstract

**Background:**

Cultivated spinach (*Spinacia oleracea* L.) is one of the most widely cultivated types of leafy vegetable in the world, and it has a high nutritional value. Spinach is also an ideal plant for investigating the mechanism of sex determination because it is a dioecious species with separate male and female plants. Some reports on the sex labeling and localization of spinach in the study of molecular markers have surfaced. However, there have only been two reports completed on the genetic map of spinach. The lack of rich and reliable molecular markers and the shortage of high-density linkage maps are important constraints in spinach research work. In this study, a high-density genetic map of spinach based on the Specific-locus Amplified Fragment Sequencing (SLAF-seq) technique was constructed; the sex-determining gene was also finely mapped.

**Results:**

Through bio-information analysis, 50.75 Gb of data in total was obtained, including 207.58 million paired-end reads. Finally, 145,456 high-quality SLAF markers were obtained, with 27,800 polymorphic markers and 4080 SLAF markers were finally mapped onto the genetic map after linkage analysis. The map spanned 1,125.97 cM with an average distance of 0.31 cM between the adjacent marker loci. It was divided into 6 linkage groups corresponding to the number of spinach chromosomes. Besides, the combination of Bulked Segregation Analysis (BSA) with SLAF-seq technology(super-BSA) was employed to generate the linkage markers with the sex-determining gene. Combined with the high-density genetic map of spinach, the sex-determining gene *X/Y* was located at the position of the linkage group (LG) 4 (66.98 cM–69.72 cM and 75.48 cM–92.96 cM), which may be the ideal region for the sex-determining gene.

**Conclusions:**

A high-density genetic map of spinach based on the SLAF-seq technique was constructed with a backcross (BC_1_) population (which is the highest density genetic map of spinach reported at present). At the same time, the sex-determining gene *X/Y* was mapped to LG4 with super-BSA. This map will offer a suitable basis for further study of spinach, such as gene mapping, map-based cloning of Specific genes, quantitative trait locus (QTL) mapping and marker-assisted selection (MAS). It will also provide an efficient reference for studies on the mechanism of sex determination in other dioecious plants.

**Electronic supplementary material:**

The online version of this article (doi:10.1186/s12864-017-3659-9) contains supplementary material, which is available to authorized users.

## Background

The spinach plant belongs to the family *Chenopodiaceae*, which is a diploid with 2n = 12 chromosomes. Spinach has a long cultivation history in China, and was introduced in the 647 A.D. Its strong resistance to cold renders it compatible with China’s short growing season. It can be multi-stubble cultivated in one year, making it an important edible green vegetable for three of the four seasons (spring, fall, and winter). Spinach is also rich in minerals and vitamins (such as vitamin C, vitamin K, calcium, iron). Because of its rich nutrition and delicious taste, spinach is one of the most popular leafy vegetables. Currently, there are more than 50 countries planting spinach, most of which are concentrated in the subtropical regions [1]. China is the largest grower and consumer of spinach. According to the Food and Agriculture Organization of the United Nations (FAO) (http://faostat3.fao.org/browse/Q/QC/E) statistics in 2014, the top three producers of spinach were China, Japan, and United States of America. China produced 287.4 million tons, which was far more than the second leading country produced 17.9 million tons. Spinach breeding and related research has aroused the attention of Chinese breeders.

Spinach is a dioecious species with separate male and female plants and occasional monoecious plants with both male and female flowers [2]. Due to its reproductive efficiency, easy cultivation, and small genome, spinach is particularly suitable for the study of the sex-determining gene [3]. For these reasons, researchers generally believe that spinach is an ideal dioecious plant model for investigating the mechanism of sex determination. In 1953, Bemis and Wilson et al. reported that sex in the species was regulated by a heteromorphic XY chromosome pair [4]. Sex has been found to be genetically associated with the longest of the six spinach chromosomes containing the alleles *X*, *X”* and *Y*, which affect sex determination [5, 6]. A reciprocal translocation proved that the sex-determining genes are located on the short arm of chromosome [7]. In 2013, the Y chromosome in spinach was isolated and analyzed by microdissection for the first time; the largest spinach chromosome was confirmed to be a sex chromosome at the molecular level [8]. Some molecular markers linked to the *X/Y* gene have been found and used for the study of molecular markers of spinach sex. Akamatsu et al. found DNAs that exist specifically in male spinach plants and then converted them to some sequence-characterized amplified regions (SCAR) markers (such as T11A and V20A). In this way, the sex of spinach can be determined easily and rapidly before bolting using this method [9]. Khattak et al. made the first genetic map of spinach, and the sex-determining gene was located on the linkage group 3 at a distance of 1.9 cM from a SSR marker called SO4 [2]. Ten amplified fragment length polymorphism (AFLP) markers and two male-specific DNAs, T11A and V20A, were mapped to a 13.4 cM region encompassing the *X/Y* locus, with an average distance of 1.22 cM between adjacent markers [10]. Though spinach is normally considered a dioecious species, monoecious plants are found in certain genotypes with various proportions of staminate and pistillate flowers [11]. The strictly monoecious character was considered to be conditioned by the known allele *X* ^*m*^ [11–13]. The genetic distance between the monoecious gene and SO4 was 4.3 cM, while the *X/Y* gene was found to be 1.6 cM with SO4. This showed that the monoecious gene was not the allele of the *X/Y* gene, but is closely linked to it [10]. Yamamoto et al. developed 19 AFLP markers and narrowed down the location of the monoecious gene. These markers spanned 38.2 cM and encompassed the monoecious gene. Linkage analysis showed that the monoecious gene and *Y* gene were located between different marker pairs. They were in different intervals [1].

In many genetic and genomic researches high-density linkage maps were valuable tools, especially for the applications of QTL mapping, map-based cloning of trait-controlled genes, and the assembly of the genome. Khattak et al. have reported a genetic linkage map using 101 AFLP and nine simple sequence repeats (SSR) [2]. The map was divided into seven linkage groups with a total length of 585 cM and the average genetic distance between the adjacent markers was 5.18 cM. It is the first time to report the construction of a genetic linkage map for spinach. Another genetic map was based on 283 Single Nucleotide Polymorphism (SNP) markers identified in actively transcribed genes with a total size of 433.6 cM [14]. The map was divided over six linkage groups that matched to the basic chromosome number in spinach, presenting a significant improvement over the published map by Khattak et al. However, some linkage groups contained relatively large gaps, 20 cM in linkage group 2 and 35 cM in linkage group 5. Due to the limitations of the research foundation, such as the lack of rich and reliable molecular markers and the shortage of high-density linkage maps, many spinach researchers were restricted in gene mapping. With the development of sequencing technology and the reduction of its cost, many species have been sequenced and resequenced. Deynze et al. have reported the reference genome Illumina sequencing work of spinach. Of those, 88% were completely assembled, and the spinach reference genome sequence was submitted to the National Center for Biotechnology Information (NCBI). This provided the basis for the follow-up study of the spinach genome.

A key section for constructing a high-density linkage map is the availability of abundant genetic markers. This is feasible due to the advances in next-generation sequencing (NGS) technology for large-scale markers discovery [15]. Several large population genotyping platforms have emerged with the practical application of NGS. SLAF-seq is based on reduced representation library (RRL) and high-throughput sequencing which provides an economical and efficient approach to large-scale genotyping. It can provide a large number of InDel and SNP markers at the same time and construct a high-density genetic map very quickly. Besides, there are considerably more markers in the genetic maps produced by SLAF-seq was than in conventional maps. This provides access for researchers to select the most information and reliable polymorphic markers needed to achieve the superior ability of functional gene mapping of important agronomic traits. SLAF-seq technology has several distinguishing characteristics that have let to its widespread use in studies in the past 3 years [16]. Up until now, this technology has been successfully applied to the construction of the genetic map in various species. Li et al. constructed a high-density genetic map for soybeans, and 41 QTLs were identified that contributed to the isoflavone content by this map [17]; Zhang et al. developed the first high-density genetic map in sesame [18]; a high-density pumpkin genetic map was constructed and identified QTLs were highly associated with pumpkin vine length [19], in Agropyron Gaertn, for wax gourds, for mei (*Prunus mume*) and so on [15, 20, 21]. More and more genetic maps are being constructed using SLAF-seq technology.

In this study, a high-density genetic map of spinach has been constructed based on SLAF-seq was reported. It is the highest density genetic map of spinach reported at present. At the same time, the sex-determining region was mapped to a single LG by the combination of BSA method and SLAF technology. This map offered a valuable tool for the study of genome assembly, QTL mapping and map-based gene cloning in spinach, and will also provide a reference for sex determination mechanism studies in other dioecious species.

## Results

### Analysis of SLAF-seq data and SLAF markers

**Fig. 1:**
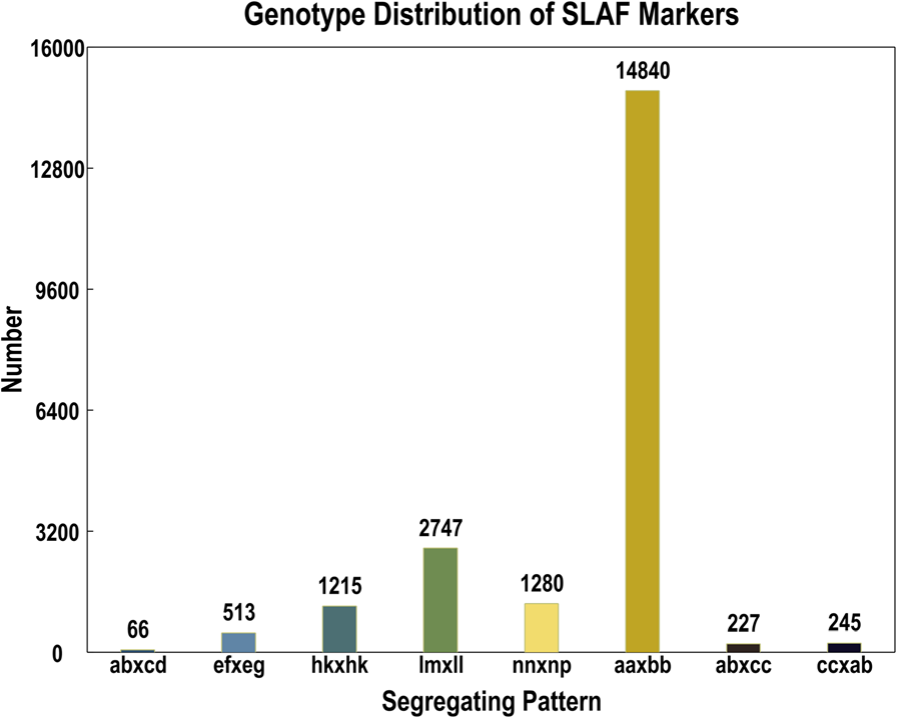
Distribution of SLAF markers in eight segregation patterns. The X-axis indicates eight segregation patterns of polymorphic SLAF markers. The Y-axis indicates the SLAF number in each pattern

Finally, a total of 207.58 million paired-end read data was obtained from the high-throughput sequencing. The Q30 percentage was 91.35%, and guanine-cytosine (GC) content was 38.51% on average. All SLAF paired-end reads with clear index information were clustered based on sequence similarity, and sequences with over 95% identity were grouped in one SLAF locus. Summary of the SLAF markers after filtering out the low, repeat, and ambiguous tags was expressed in detail in Table 1. As shown, the SLAF’s number of maternal and paternal parents was 124,866 and 111,226, with an average of 34.39-fold and 31.31-fold coverage, respectively, while the SLAF number of offspring was 83,812, with an average depth of 7.22-fold coverage. Through the statistical analysis of SLAF tag data of parents and offspring, 145,456 different high-quality SLAFs were developed altogether, and only 27,800 of the SLAF’s tags were effective polymorphic SLAFs, with a polymorphism percentage of 19.11% (Table 2). In order to facilitate subsequent genetic analysis, molecular markers were needed to carry out genotype encoding with genetics’ two allelic general encoding rules as shown in Table 3. Finally, 21,133 of the polymorphic SLAF tags were successfully encoded. Genotype distribution of SLAF markers are shown in Fig. 1. A total of 14,840 SLAFs fell into the aa × bb segregation pattern which were used for genetic map construction, the effective polymorphism of the genetic map was 10.20%. To ensure the quality of the genetic map, SLAF tags were filtered with some rules, which are shown in the method details. Finally, 4,092 SLAFs were obtained and used for the construction of the linkage map.

**Table 1:** Summary of the developed specific length amplified fragment (SLAF) markers

Samples	SLAF number	Total depth	Average depth
Maternal parent	124,866	4,294,036	34.39x
Paternal parent	111,226	3,482,252	31.31x
Offspring	83.812	598,517	7.22x

**Table 2:** Polymorphism analysis results of the SLAF markers

Type	Polymorphic SLAF	Non-polymorphic SLAF	Repetitive SLAF	Total SLAF
Number	27,800	116,814	842	145.456
Percentage (%)	19.11	80.31	0.58	100.00

**Table 3:** Genetic encoding rules

Type	Paternal genotype	Maternal genotype	Offspring genotype
abxcd	ab	cd	ac,ad,bc,bd,--
efxeg	ef	eg	ee,ef,eg,fg,--
abxcc	ab	cc	ac,bc,--
ccxab	cc	ab	Ac,bc,--
hkxhk	hk	hk	hh,hk,kk,--
lmxll	lm	ll	lm,ll,--
nnxnp	nn	np	nn,np,--
aaxbb	aa	bb	aa,bb,ab,--

### High-density genetic linkage map construction for spinach

**Fig. 2:**
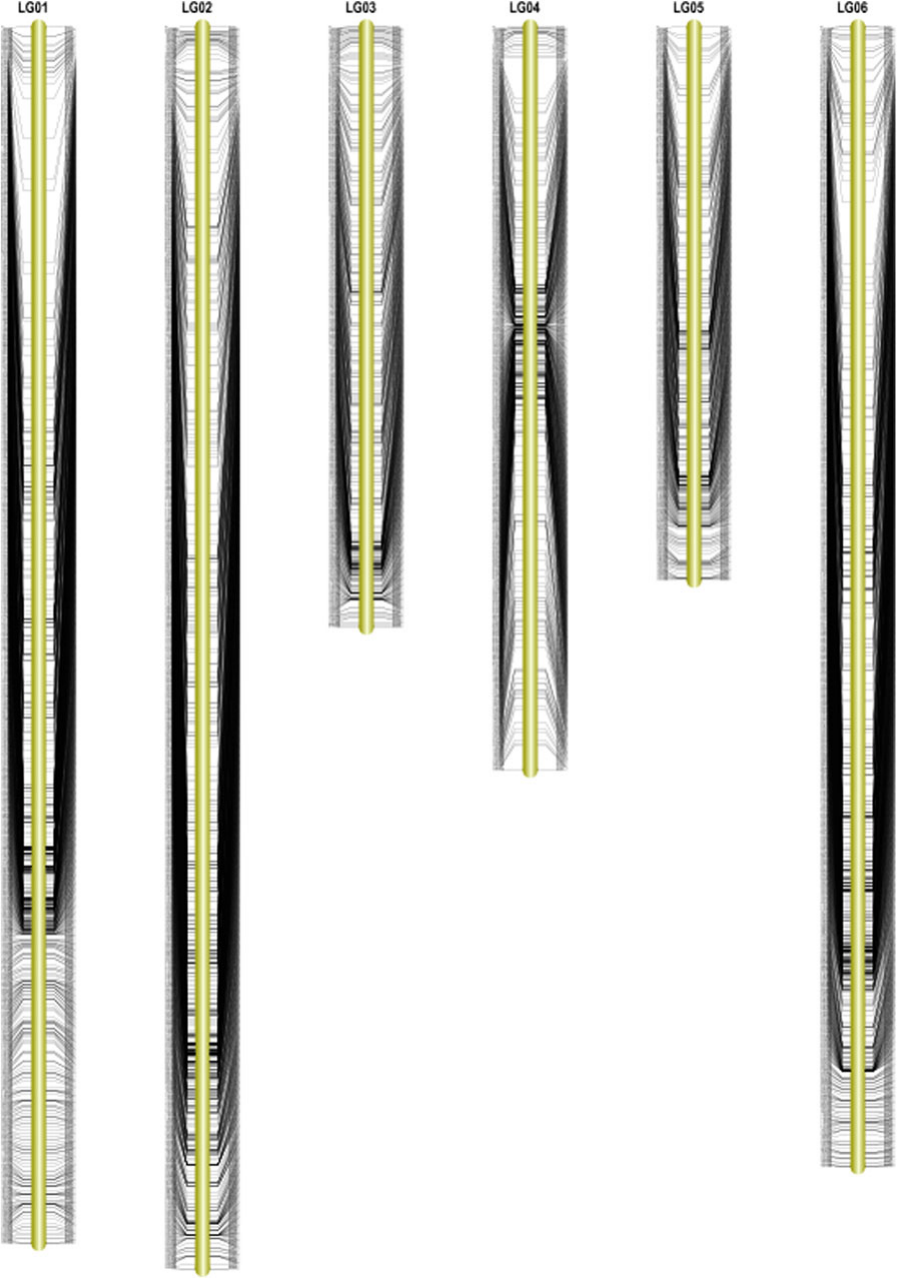
Distribution of SLAF markers on 6 linkage groups of spinach. SLAF marker names and their locations are listed on the right and left sides of the axis

A high-density genetic map of spinach based on SLAF-seq technology was ultimately constructed. After linkage analysis, 4,080 SLAF markers were mapped onto the genetic map, making the mapping ratio of the available SLAF tags about 99.71%. With different linkage groups, the distribution of SLAF numbers ranged from 411 in LG5 to 922 in LG2 (Table 4). The map spanned 1,125,97 cM with an average genetic distance of 0.31 cM between the adjacent marker loci (Fig. 2). It was divided into 6 linkage groups corresponding to the number of spinach chromosome. The length of the linkage groups in the genetic map ranged from 178.66 cM (LG6) to 201.28 cM (LG3), with an average of 187.66 cM. LG1 and LG2 was the highest density group, containing 903 and 922 markers, respectively, both with an average marker density of 0.2 cM. The shortest linkage group was LG6 that contained 846 markers with an average genetic distance of 0.21 cM. The longest linkage group was LG3, which contained 446 markers with an average genetic distance of 0.45 cM. The largest gap, which indicated the degree of uniformity, was located on LG1and LG6 with a marker interval of 10.94 cM. To our knowledge it is the highest density genetic map of spinach reported at present.

**Table 4:** Description of basic characteristics for the six linkage groups

Linkage group ID	Total marker	Total distance (cM)	Average distance	Max gap	SNP number
LG1	903	184.18	0.2	10.94	1,329
LG2	922	187.43	0.2	3.95	1,361
LG3	446	201.28	0.45	4.44	602
LG4	552	194.01	0.35	10.38	798
LG5	411	180.41	0.44	7.31	576
LG6	846	178.66	0.21	9.78	1,200
Total	4080	1,125.97	0.31	10.94	5,866

The integrated genetic of spinach contained 3 types of molecular markers: ‘SNP-only’-type, ‘InDel-only’-type, and ‘SNP&InDel’ markers, of which ‘SNP-only’ was the main type. There were a total of 5,866 SNP markers in the genetic map. Table 4 shows the number distribution of SNP markers. SNP mining and typing is an important step in gene mapping. SNPs are the more appropriate choice for genetic analysis based on sequencing at present; it has many advantages, such as large number, wide distribution, easy genotyping, and suitability for rapid and large-scale screening. These SNP markers might provide important information for the location of functional genes.

### Mapping of the sex-determining gene *X/Y*

**Fig. 3:**
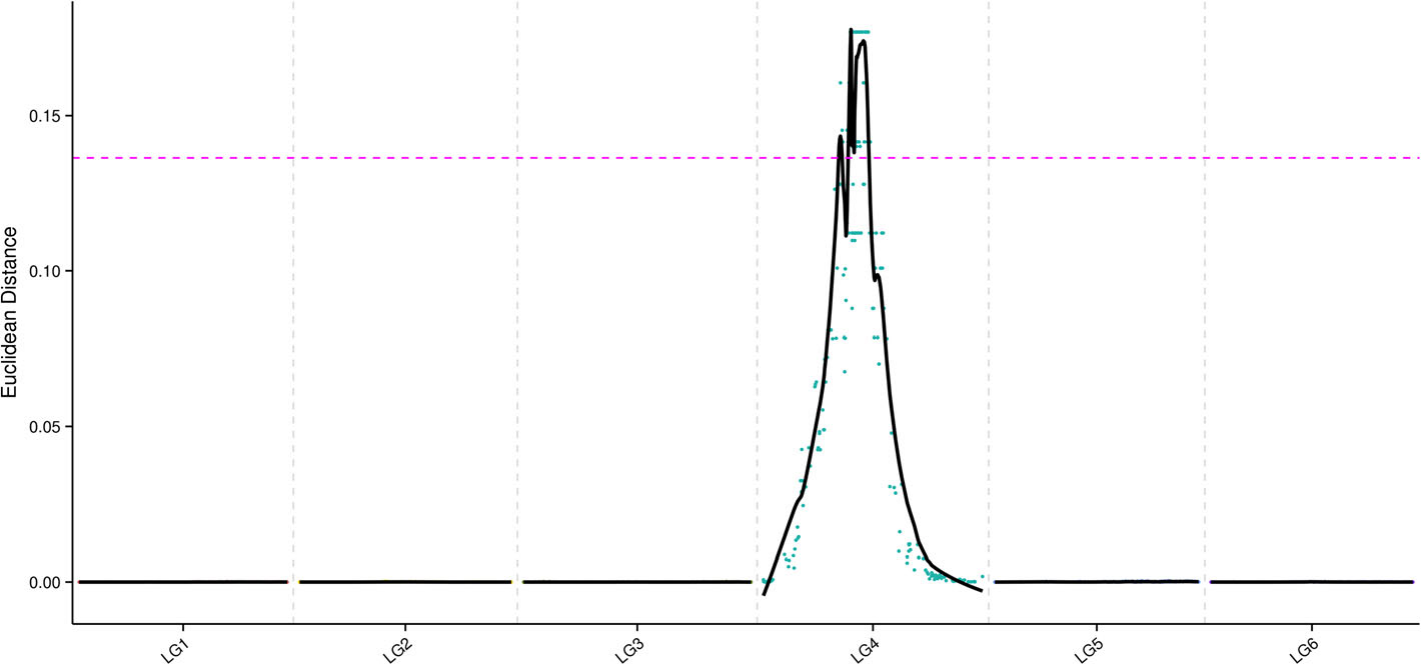
Analysis of the sex phenotype on linkage groups (the x-axis indicates linkage groups; the y-axis indicates ED value; the red line is the threshold value)

To illustrate the sex determination of spinach, we used super-BSA to identify candidate genes associated with sex in spinach. Combined with the high-density genetic map of spinach, 323 associated SLAF markers were identified, and these markers were concentrated in two hot spots, which contained 166 genes on LG4 that may be related to the sex differentiation of spinach (Fig. 3) (Table 5). Position results showed that the sex determination region (SDR) were fine mapping to the corresponding chromosome, which will provide important information for further studies of spinach (e.g., sex determination gene cloning, molecular expression mechanism, and functional studies).

**Table 5:** Information of the association region

Linkage group	Start	End	Size (cM)	Diff_marker number	Gene number
LG4	66.98	69.72	2.73	45	23
LG4	75.48	92.96	17.48	278	143

## Discussion

### High-density genetic map application

There were only a few studies on the genetic map of spinach, and, until now, only two genetic linkage maps were published. One study was constructed in a classical backcross population with 110 markers of SSR and AFLPs [2]. The markers number of this genetic map was not big enough, and the genetic map was divided into seven linkage groups,since spinach has only six chromosomes, two of the present linkage groups should probably be joined. The other genetic map was based on 283 SNP markers identified in actively transcribed genes with a total size of 433.6 cM [14]. The map was divided over six linkage groups, have more number and more convenient marker over the published map by Khattak et al. However, the markers in linkage group 2 and in linkage group 5 were limited and not evenly distributed. In this study, a high-density genetic map of spinach based on SLAF-seq technique was constructed. Here, 4080 SLAF markers were located on the genetic map. The map spanned 1,125.97 cM and was divided into six linkage groups corresponding to the number of spinach chromosomes, with an average distance of 0.31 cM between the adjacent marker loci. Compared with the two previously reported genetic maps, this map has more numbers for accurate gene mapping. Finally, the traditional method of constructing a genetic map was very time consuming and laborious; restrictions in throughput are a limiting factor of large-scale genotyping in the construction of a high-density genetic map.

NGS has revolutionized genomics, which allows efficient development of a large number of polymorphic markers in a short time and enables high-density genetic map construction to be more efficient than ever before. With the increase of the sequencing level and the reduction of the cost of sequencing, more and more high-density genetic maps were constructed. These were exceptionally valuable tools for QTL mapping, fine-scale mapping, and map-based cloning of trait-controlled genes. With a high-density genetic map of glycine max, the consistent QTLs for isoflavone content across various environments were identified [17]. Liu et al. used 1712 SSR primer pairs to construct a map, followed by the identification of yield and seed trait QTL in upland cotton [22]. A locus-controlling weeping trait in Mei was identified by the application of the high-density of genetic map [15]. One QTL for walnut anthracnose resistance was identified based on the high-density genetic map of walnuts [23]. A high-density genetic map of pumpkins was constructed for anchoring genome sequences and identifying QTLs associated with the dwarf vine [19]. These successful localizations of important agricultural traits strongly indicate that the high-density map is a worthy reference in many genetic and genomic applications.

### The advantage of SLAF and super-BSA

The SLAF represents a revolution in the simplified genome sequencing technology by its special characters, such as its effective read length, high throughput, flexible program design, and so on. It finds the best balance between the cost of sequencing and the depth of the sequencing. SLAF can obtain the whole genome-wide variation image (SNPs, InDels) and provide access for researchers to choose the most informative and reliable polymorphic markers in order to achieve the functional gene mapping of important agronomic traits of excellence. BSA was commonly used to locate two extreme-trait-related genes or regions. It is a classical method for rapidly and effectively identifying markers target genes, and it is widely used in crop breeding studies. Super-BSA combined the SLAF-seq with BSA to identify candidate regions associated with target traits. Super-BSA has the following advantages over traditional BSA:

i. Large-scale mixed pool - The number of individuals in the mixed pool could reach or exceed 200, which is larger than the traditional BSA.
ii. Higher marker density : The number of sequenced tags that were used for DNA pools scanning can reach 100,000.
iii. Quantitative statistical genotype frequency - The use of second-generation sequencing technology provided direct access to the digitized signal.
iv. Finer positioning: The SNPs obtained from SLAF-seq is evenly distributed in the genome and repetitive regions were avoided to the greatest extent possible, so the positioning effect was better than a traditional gel-based marker.
v. Cost-effectiveness - Uses the same cost with traditional marker screening and there is no need to invest excessive amounts of manpower and time.

In this study, we used sequencing data instead of DNA samples to produce the extreme trait pool based on the spinach sex phenotype. Each association marker was supported by the individual plant data, which would facilitate follow-up studies. Super-BSA may greatly speed up the progress of target trait mapping and molecular marker-assisted selection breeding in the future.

### The follow-up study of the sex-determining gene mapping in spinach

Spinach sex is controlled by the sex-determining gene *X/Y*. Akamatsu et al. developed two SCAR markers, T11A and V20A, which lined with the *X/Y* gens [9]. In the first genetic map of spinach made by Khattak et al., the *X/Y* gene was mapped on LG3 with a distance of 1.9 cM to SO4 [2]. Ten AFLP markers developed by BSA method were closely linked to the *Y*, in which four markers were co-segregate with *Y.* Combined with T11A and V20A, the markers were mapped to a 13.4 cM region encompassing the *Y* locus, with an average distance of 1.22 cM between adjacent markers [10]. Yamamoto et al. developed 19 AFLP markers closely linked to the monoecious gene, and suggested that the monoecious gene and *Y* were located in different intervals, T11A and V20A were co-segregated with *Y* and SO4 was lined with the monoecious gene. T11A and V20A markers were used in all these studies. In the current study, the super-BSA method was used to identify regions associated with the sex-determining gene of spinach. Ultimately, two hot regions with 323 markers linked to the sex-determining gene were obtained, the sex-determining gene *X/Y* was located at the position of LG4 (66.98 cM–69.72 cM and 75.48 cM–92.96 cM), which were supposed to be the sex determining regions. The markers T11A and V20A have been used to detect the mapping population. Results showed that the two markers were also located in the mapping region and co-segregated with *X/Y* gene. So our *X/Y* gene mapping region was consistent with previous research results and showed a finer accurate positioning.

Using the genetic map to assist the spinach reference genome assembly, the scaffold was anchored to the corresponding sex linkage markers. Through bioinformatics analysis, there were 166 genes in the two hot regions associated with sex determination. The function of these genes will be studied further to elucidate the mechanism associated with the sex-determining gene in spinach, and the associated SNPs will be used for MAS of spinach. The plan for the next work is to expand the segregation population and develop new SSR, InDel, and SNP markers for fine mapping of the sex-determining genes. This is the first time any study, domestic or international, has used spinach reference genome information combined with the rapid development of high-throughput sequencing technology to study the molecular mechanism and cloning of the sex-determining gene of spinach. This work also provides reference data for the further study of other characters of spinach.

## Conclusions

A high-density genetic map of spinach based on the SLAF-seq technique was constructed, and is currently the highest density genetic map of spinach reported. In addition, the high-density genetic map of spinach was combined with super-BSA to identify candidate genes associated with sex in spinach. Finally, 323 associated SLAF markers were identified, and two main hot regions that were detected on LG4 may relate to the sex differentiation of spinach (Fig. 3); these contained 166 genes in the two regions. The use of this map will lay the foundation for the study of the molecular biology of spinach, and may be applied toward additional research endeavors (e.g., gene mapping, map-based cloning of genes, marker-assisted breeding, etc.). It will also provide a reference for studies in other dioecious plants and assist in studying the mechanism of sex determination.

## Methods

### Plant material and DNA extraction

The mapping population included two parents and 148 BC_1_ individuals that were derived from a cross between an inbred line 12S4 as the male parent, and an inbred line 12S3 as the female and recurrent parent. The 12S4 line, which was characterized by dark green leaves, a narrow blade, and a long petiole, while 12S3 had light leaf color, a wide blade, and a short petiole. Both of the inbred were dioecious plants and no monoecious plants were found in the offspring. All of the materials were cultivated in the spring of 2015 in a natural environment in the experimental field of the Institute of Vegetables and Flowers, Chinese Academy of Agricultural Sciences. The fresh and tender leaves were collected from the 148 individuals and their parents, and then frozen in liquid nitrogen to prepare for genomic DNA extraction. Total genomic DNA was extracted according to the cetyltrimethyl ammonium bromide (CTAB) method [24]. DNA concentration and quality were estimated with an ND-2000 spectrophotometer (Thermo Fisher Scientific, Wilmington, DE, USA) and by electrophoresis in 1.0% agarose gels with 5 K marker (Trans Gen Biotech). By testing, the concentration and quality of all samples were qualified, and could be used in the construction of the SLAF library.

### SLAF library construction and high-throughput sequencing

Spinach-1.0.3 was selected as the reference genome for prediction of enzyme digestion according to genome size and GC content. It is the latest updated version and can be downloaded from http://www.ncbi.nlm.nih.gov/genome/7547?genome_assembly_id=240834. The estimated genome size of spinach is 956 Mb, while the size of the assembled genome that we finally used was only 493.771 Mb, and the GC content was 38%. A pre-design SLAF experiment was performed to predict the results of enzyme digestion of the reference genome and select the optimum enzyme digestion method. Four criteria were considered in the selection: i) The suitable restriction site was located as less in the repetitive sequence as possible; ii) The enzyme fragment was uniformly distributed in the genome; iii) The length of the fragments produced by double enzyme digestion were consistent with the specific experimental system; iv) The number of SLAFs must met the expected number of labels. These considerations improved the efficiency of the SLAF-seq. According to the results of predesigned scheme, two enzymes were selected to completely digest the qualified genomic DNA separately. After complete digestion, the ends of different fragments were repaired to blunt-ended DNA and the 5′ends were phosphorylated. An adenine was added to the 3′end of the digested fragment and duplex tag-labeled sequencing adapter was then ligated to the A-tailed fragment. The DNA fragments from different individuals were attached to different adapters so all samples could be sequenced in parallel to achieve the purpose of high-throughput sequencing. In order to make the concentration of fragments to meet the sequencing requirements, 20 polymerase chain reaction (PCR) cycles were performed to enrich the DNA products. Forward primer: 5′-AATGATACGGCGACCACCGA-3′, reverse primer: 5′-CAAGCAGAAGACGGCATACG-3′ (PAGE-purified, Life Technologies). PCR products were then purified and pooled. The separation of pooled samples was performed by 2% agarose gel electrophoresis (120 V, 60 min). In accordance with the prediction results of enzyme digestion, fragments ranging in size from 364 to 414 base pairs were the target DNA bands. The gel-purified products were diluted to equal concentration after excision and purification. Subsequently, an Illumina HiSeq 2500 (Illumina, Inc; San Diego, CA, U.S.) system was used for pair-end sequencing (each end 125 bp) as recommended by the manufacturer. SNP genotyping and evaluation were then performed.

### SLAF-seq data grouping and genotyping

SLAF marker identification and genotyping were performed using procedures described by Sun et al. with minor modifications [16]. Real-time monitoring was performed to control the quality of the sequencing data in each cycle during the sequencing progress. Two key indicators were calculated, sequencing quality value (Q) and guanine–cytosine (GC) content. In the process of base calling, a sequencing quality value was obtained for each base, which was used to evaluate the accuracy of the base. Q is an important indicator in evaluating the high throughput single base error rate (e). The corresponding formula: Q = −10 × log10^e^. The higher the value of Q, the lower the error rate of base. We used Q30 value (which means the probability of an error in a base sequence was 0.1%) to evaluate the sequencing quality of the SLAF tags. Low-quality reads with a score of < Q30 were filtered out. The SLAF tags were clustered based on the sequence homology detected by BLAT (-tileSize = 10 -stepSize = 5) [25]. Sequences with over 95% identity were grouped in one SLAF locus as described by Sun et al. [16]. To obtain good quality SLAF tags, these groups were filtered out in the following conditions after clustering: i) low tags: parental sequencing depth below 10x or sequencing errors led to only matched reads; ii) repeat tags: because spinach is a diploid species, one locus contains four SLAF tags at most, so groups containing more than four tags were filtered out as repeat SLAFs; iii) ambiguous tags: a wide range of reads numbers for tags in one group led to ambiguous, and these SLAFs were also filtered out. After filtering, SLAFs were classified according to the number of tags in each group. By analyzing the polymorphism of SLAF tags in the parents, the tags were divided into three types: polymorphism SLAFs, Non- polymorphism SLAFs and Repetitive SLAFs. SLAFs with more than two tags in one parent were defined as Repetitive SLAFs and were filtered out; those with only one tag in groups were defined as Non-Polymorphic SLAFs and were also filtered out; SLAFs with two, three, or four tags were identified as polymorphic SLAFs and considered to be potential markers suitable for subsequent analysis. The polymorphic markers were classified into eight segregation patterns by using two allele coding rules in genetics (ab × cd, ef × eg, hk × hk, lm × ll, nn × np, aa × bb, ab × cc, and cc × ab). The mapping population was a BC_1_ population, so tags in aa × bb segregation patterns were used for genetic map construction.

High-quality SLAF markers for the genetic mapping were filtered by the following criteria: i) Filtered parental DNA sequencing depth below 10x; ii) Integrity below 60%; iii) SNP more than 7; iv) significant segregation distortion (P < 0.05) of polymorphic SLAFs**.** Only those polymorphic SLAFs with appropriate sequencing depth were left to perform the follow-up analysis.

### High-density genetic map construction

A newly developed strategy HighMap was employed to order the SLAF markers and correct genotyping errors within LGs [26]. According to the protocol detailed by Liu et al., the HighMap strategy contained four modules. First, it was designed to include a linkage grouping module: marker loci were partitioned primarily into LGs based on their locations on the spinach genome. Next, the modified logarithm of odds (MLOD) scores between markers was calculated to further confirm the robustness of markers for each LG. Markers with MLOD scores <5 were filtered prior to ordering. Second, the marker ordering module was designed: the enhanced Gibbs sampling, spatial sampling, and simulated annealing algorithms were combined to conduct an iterative process of marker ordering [27, 28]. Third, error genotyping correction module was established using SMOOTH strategy to conduct the error genotyping correction according to parental contribution of genotypes [28], and a k-nearest neighbor algorithm was used to impute missing genotypes [29]. In order to produce better results, the ordering module and error genotyping correction module were carried out iteratively. Finally, the map evaluation module was established to evaluate the quality of the genetic map. Map distances were estimated using the Kosambi mapping function.

### Mapping of the sex-determining gene *X/Y* with super-BSA

Super-BSA method was used to illustrate the sex determination of spinach [30]. The mapping population was divided into 2 groups using a sex phenotype, and then SNPs were obtained from SLAF tags on the genetic map in different groups. SNPs were generated via sequencing of mixed pools to conduct data analysis and generate the linkage markers with the sex-determining gene. In this study, we chose to measure allele segregation using Euclidean distance (ED) as a metric to identify the region associated with sex [31]. ED was calculated at each SNP location using the following equation:

$$ E D=\sqrt{{\left({A}_F-{A}_M\right)}^2+{\left({C}_F-{C}_M\right)}^2+{\left({G}_F-{G}_M\right)}^2+{\left({T}_F-{T}_M\right)}^2}. $$

Here, each letter (A, C, G, and T) corresponds to the frequency of its corresponding DNA nucleotide. F and M represent the female and male characters, respectively. Theoretically, there was no difference between the two pools in addition to the target trait loci, and therefore the ED value of the non-target site would tend to be 0. The greater the value of ED, the greater the difference between the two pools. ED is advantageous because it is linear, making it less prone to errors in allelic frequency analysis, and it can subtract out sequence-specific errors, such as artifacts of Illumina sequencing technology, which are assumed to be present at equal frequency in both samples [32]. It does not require parental strain information, and it is resistant to noise. To calculate the correlation value using the above ED method, we first took the original ED 5 party as the correlation value to eliminate background noise. We used the local linear regression LOESS method to fit the ED value and took all of the sites fitted values mean + 3SD as an analysis of the associated threshold and according to the correlation threshold. The region above the threshold value was selected as the region associated with the trait [33].

## Additional files

Additional file 1: Genotype for BC_1_ individuals. (XLSX 2427 kb)

Additional file 2: High-density genetic linkage map of spinach constructed in this study. (XLSX 100 kb)

Additional file 3: The SLAF markers encompassed in the sex-determining gene locus as revealed by SLAF-BSA analysis. (XLSX 25 kb)

## Abbreviations

AFLP: Amplified fragment length polymorphism
BC_1_: Backcross
BSA: Bulked segregation analysis
CTAB: Cetyltrimethyl ammonium bromide
ED: Euclidean distance
FAO: Food and agriculture organization of the United Nations
GC: Guanine-cytosine
LG: Linkage group
MAF: Minor allele frequency
MAS: Marker-assisted selection
MLOD: The modified logarithm of odds
NCBI: National Center of Biotechnology Information
NGS: Next-generation sequencing
PCR: Polymerase chain reaction
QTL: Quantitative trait locus
RRL: Reduced representation library
SCAR: Sequence characterized amplified regions
SDR: Sex determination region
SLAF-seq: Specific-locus amplified fragment sequencing
SNP: Single nucleotide polymorphism
SSR: Simple sequence repeats
super-BSA: SLAF-seq combine with BSA
